# Address of Francis S. Colley, M. D., Late President of the Medical Association of Georgia, upon Retiring from the Chair

**Published:** 1860-05

**Authors:** 


### ARTICLE III.

*Address of Francis S. Colley, M. D., late President of the Medical Association of Georgia, upon retiring from the Chair.*

Gentlemen of the Medical Association—

I am now about to yield the Chair, to which your kindness elevated me, to the worthier occupancy of my successor. Permit me, before retiring, to call your attention to certain causes that conspire to lower the standard of the Medical Profession. Our ranks are full to overflowing. Each year brings additional members in a ratio of increase beyond that of the population and wants of the country. A cursory glance at the different classes of Physicians, will render manifest the mischiefs to which I allude. The membership of no

Profession possess more varied intellects and attainments than do we of the Medical. Some of the profoundest minds of every age have illuminated our walks. These are not many, as the number of investigators after hidden truth in science, has ever been small.

Such is man's proneness to adopt, as the only truths capable of being evolved, the dicta of predecessors, that the greater number of truly gifted minds rust in indolent acquiesence in the theories of others. Few, however colossal in intellect, have the nerve to elaborate and promulgate opinions that conflict with the orthodox tenets of Medicine. Happily for the human race, there are pioneer intellects who disdain the trammels of a blind devotion to the canons of whatever master, and investigate for themselves. Their crude deductions are laughed at, and sneered at; but when, better developed, they gain, inch by inch, a foot-hold in the vestibule of the temple of Medicine, the scoffers cease their cavilling, and are the first to fling up their hats to honor the inauguration of the strange doctrines within the very temple itself.

I congratulate you, Gentlemen, that there are in our midst persons of this class, who are carving for themselves reputations more durable than brass or marble. Their names will be reverenced as long as science maintains her supremacy in the halls of Medicine.

Another class of Physicians, much larger than the former, trouble their heads very little about scientific generalizations. The arcana of nature remain such to them, until seen through the eyes of others. By close observation during years of laborious practice, they learn to apply readily the remedies the text-books teach, to the diseases the text-books describe. The utmost they venture is to report anomalous cases as data for the speculations of their more daring brethren. This class of routine practitioners must not be despised—they are the "bone and sinew" of the profession. Making no pretensions to genius, they stand between mankind and the theorists, by filtering the speculations of the latter through common sense.

Everybody can understand the difference betwixt genius and common sense. Genius dreams divers remarkable dreams and originates many beautiful theories. But don't you see that genius is powerless to do aught save to speculate and dream, until aided by common sense? Its very success becomes in part the success of common-sense. On the other hand, common sense can accomplish just as little, until genius shall have furnished the wherewithal. So the class of whom I am speaking are the practical working men, who *originate* nothing, but *adapt* everything to the requirements of the race.

I regret to add that there is a third class of Physicians scarcely less numerous than the last. It would be well for the fraternity if no such class existed. They contribute much to fix the tone of character of the entire profession, as recognized amongst the masses. They abound everywhere.—They may be known by their impudence, loquacity and braggardism. They have the rare luck to be always pressed with practice, albeit they employ a deal of clap-trap to convince others of the fact. They have grappled, according to their frequent assertions, with every disease known to the books, besides many diseases that are *not known* to the books. They are apt to disregard the regular fee-bill, and to fight their rivals with the dishonorable weapon of low charges.—They sometimes subscribe for the Journals, but rarely read, and *never* pay for them. Study they disdain; prefering to gather by absorption the scanty knowledge they acquire after entering upon the practice. They are the quacks that curse society; that contrive to eke out a disreputable subsistance at the expense of credulous patrons. Such men resort to the getting up of tinctures, pills, and other empirical compounds for the cure of specific diseases. They invariably append to their names the talismanic abbreviations, M. D., which, by the way, are fast becoming the exclusive property of Charlatans.

Who are responsible for this excrescence upon our noble profession? I dare say each one of you has asked the same question a score of times. Who, but the profession at large, are responsible? These pretenders in medicine are instructed in our offices and graduated in our colleges. The practice prevails, when a boy has not the business shrewdness and energy to become a merchant, or the learning and talent to become a lawyer, or the industry to become a farmer, or the piety to become a preacher, to hasten him, after a few month's idling in the nearest office, to the remotest Medical College, to be returned from thence a galvanized doctor.

The Colleges are not wholly to blame in this matter. That the course of instruction is not as full as it should be, cannot be denied. Nor can the fact be concealed, that a majority of the students evade the greater part of the duties actually required at their hands. If we send incompetent students from our offices to the Colleges, they will invariably return incompetent graduates. Badly instructed at home, they go there to fall into the hands of "quizzers" or "grinders," and to practice upon the Faculties the first lessons of deception and quackery, which they afterwards practice upon their unfortunate patrons. The reformation must begin, if at all, with the individual members of the Profession. Let the office-preceptors close their doors upon all applicants, who are not known to possess respectable talents, moral character, studious habits and competent scholarship. The absence of these qualities unfits any man to be an efficient physician.—A collegiate education would be much the best, as in that case, habits of study would ordinarily be fixed. Coming possessed of these requisites, let the student remain two or more years, diligently mastering the elementary branches.—When he has accomplished this, let him receive from his preceptor a certificate of fitness to enter a Medical College, without which certificate the Colleges should matriculate no applicant. Let the College course be extended to three terms or courses of Lectures. Suppress all "quizzing" or "grinding" classes, and substitute thorough written examinations for graduation, instead of the inefficient oral examinations.

This course of reform may be considered too rigid, but the necessity of the case requires something rigid in order that it be effectual. Nor is it open to the objection that the deserving poor would be excluded from the path of Medicine. The day is not far distant in Georgia, when all classes will be educated at public expense. Besides, merit will always insure to the poor, friends to help them. I would also call your attention specially to the prevalent disregard of Medical Ethics. Nine-tenths of the bickerings that disgrace the profession, spring from an ignorance of this important subject. Physicians might learn from the Gentlemen of the Legal Profession, a valuable lesson in the courtesies that should characterize brethren.

Many of us, Gentlemen, have attended upon the Lectures of Northern Colleges. We should never suffer a student to follow our example. We should resolutely set our faces against the practice, in future, of neglecting our own flourishing institutions.

Permit me, Geutlemen, to again tender you my sincere thanks for the honor you have conferred upon me, and for the courteous assistance you have rendered towards the discharge of the arduous duties which I now lay aside.

